# Neural correlates of consciousness in the sound-induced flash illusion

**DOI:** 10.1038/s41598-025-28068-5

**Published:** 2025-11-28

**Authors:** Theresa Rieger, Josefine Feuerstein, Niko A. Busch, Thomas Straube, Maximilian Bruchmann

**Affiliations:** 1 Institute of Medical Psychology and Systems Neuroscience, University of Münster, Von-Esmarch-Str. 52, D-48149 Münster, Germany; 2 Otto Creutzfeldt Center for Cognitive and Behavioral Neuroscience, University of Muenster, Muenster, Germany; 3 Institute of Psychology, University of Muenster, Muenster, Germany

**Keywords:** NCC, EEG/ERP, Visual awareness, Consciousness, VAN, Illusions, Neuroscience, Psychology, Psychology

## Abstract

The visual awareness negativity (VAN) has been identified as a potential neuronal correlate of consciousness (NCC). The VAN is typically found when comparing experimental trials in which a stimulus was perceived with trials in which the same stimulus was not perceived. However, if the VAN represents a reliable NCC, it should also be observed under conditions in which participants report conscious perception despite the absence of a corresponding visual stimulus, i.e., a visual illusion. Our event-related potential (ERP) study (*N* = 47) aimed to investigate this question by using a suited stimulation design, the sound-induced flash illusion (SIFI), for which a visual stimulus is presented once together with two short beeps, leading - on about half the trials - to the illusory perception of a second flash, with graded levels of reported awareness of these trials. When comparing illusory with non-illusory trials, we found an enhanced negativity over posterior electrodes between 250 and 300 ms. Frequency analyses additionally revealed lower pre-stimulus alpha power in illusion trials, aligning with prior work linking alpha dynamics to perceptual variability. Our findings suggest that even illusory perceptions enhance negative potentials over posterior regions during the VAN interval, supporting the interpretation of the VAN as a NCC.

**Supplementary Information:**

The online version contains supplementary material available at 10.1038/s41598-025-28068-5.

## Introduction

 The neural correlates of consciousness (NCC) refer to the basal neural mechanisms that, in combination, are sufficient “for any one specific conscious experience"^1^. In recent years, substantial advances in consciousness research have been achieved, e.g., through studies utilizing electroencephalography (EEG), which suggest potential NCC candidates^2–4^. EEG studies on the neural correlates of visual consciousness typically make use of a mismatch between the visual stimulation and the following perception. Most frequently, this approach involves a “hit vs. miss” comparison, i.e., the comparison of trials in which a stimulus was reported as seen, with trials in which the same stimulus was reported as unseen. Based on this approach, an enhanced negative event-related potential (ERP) emerging approximately 100–300 ms after stimulus onset has been identified; this negativity, known as the Visual Awareness Negativity (VAN), is most prominent over occipital and posterior temporal scalp sites^5–9^. The VAN is claimed to be the earliest and most reliable marker of visual consciousness (for review see^3^) and is in accordance with consciousness theories, which posit a central role of early sensory areas in generating conscious perception of a stimulus^4,10,11^. Previous research shows that a negative deflection is not only measurable in visual perception but also in other sensory modalities. In this framework, the VAN is part of a family of components of perceptual awareness negativities (PAN^2,12^) with an equivalent in the somatosensory modality (somatosensory awareness negativity, SAN^13,14^) and the auditory modality (auditory awareness negativity, AAN^15–18^).

Yet, the role of the VAN as a direct marker of consciousness remains debated. Conscious access has been associated either with both the VAN and subsequent late positive components^19^ or with later positive components alone^20–24^. In this framework, the VAN may reflect early, pre-conscious processing, potentially indexing preparatory or unconscious perceptual activity rather than awareness itself^25,26^. If VAN-like negativities also occur without external input, such as during illusory perception, this would challenge their interpretation as purely pre-conscious and support their role in conscious awareness. Illusory percepts thus offer a strong test case for disentangling stimulus-driven activity from awareness-related neural markers, as they show (along with vivid dreams^4^ and other forms of hallucinations^27,28^) that the production of percepts by the brain does not depend on the bottom-up (feed-forward) processing of sensory information^11^.

In predictive coding frameworks, the VAN may also index a prediction error, i.e., the mismatch between the expected and the actual stimulation^29^. In the typical hits vs. misses comparison, a seen target could be explained by the strong expectation of its absence (e.g., an empty display) and a stimulus signal that is sufficiently surprising^30^ to trigger an update of the expectation, leading to a veridical perception. Misses, however, would be the result of an insufficient error signal, causing perception to be dominated by the predicted absence of the stimulus. An increased negativity for seen vs. unseen targets could therefore correspond to both the conscious perception of the target or the prediction error leading up to it. Illusion paradigms offer the possibility to dissociate these interpretations, because conscious perception would be the result of a small prediction error, leading to the expected perception of a stimulus in the absence of actual stimulation. If the VAN indexes a prediction error, it would therefore be smaller for illusions compared to the veridically perceived absence, whereas it would be larger if it indexed conscious perception.

We examine the question of whether the VAN is exclusive to comparisons between seen and missed veridical stimuli, or whether it can also emerge when contrasting illusory percepts with correctly perceived stimulus absence. To our knowledge, only few studies have directly examined early ERPs elicited by consciously perceived but physically absent stimuli. In the auditory domain, studies have either elicited illusions with Pavlovian conditioning^31^ or relied on spontaneous auditory illusions occurring in noise^32^. In both studies, negative deflections were more pronounced for illusory percepts, consistent with the AAN^31,32^. One study also observed a late positivity for illusory percepts, however, interpreted as centro-parietal positivity (CPP), suggesting a decision-related or higher-order cognitive process^32^. For the visual modality, several studies have examined illusory flashes, triggered by the simultaneous presentation of stimuli in other modalities. With auditory trigger stimuli, this phenomenon is called the Sound-Induced Flash Illusion (SIFI^33,34^, see^35^ for a Touch-Induced Flash Illusion, TIFI). The SIFI occurs when a single visual flash is accompanied by two auditory beeps, leading participants to perceive a second, non-existent flash^33,36^. Building designs that produce convincing ERP results in the illusory domain is inherently challenging. Illusion rates must be sufficiently high and yield enough trials for statistically valid averages. Equally crucial is precise timing because the onset of an internally generated percept is difficult to define, other than in paradigms where the onset of a perception trigger is identical with the onset of an external stimulus. The SIFI not only produces a comparably high rate of illusory perceptions in stimulus-absent trials (around 50%^37^) but also enables precise estimation of the perceptual onset, as the illusory second flash coincides subjectively with the onset of the second beep. This inference of the illusory percept’s onset al.lows for investigating ERPs associated with visual illusions. The SIFI is widely used to study multisensory integration (for review see^38,39^), but few studies have compared ERPs between illusion and non-illusion trials^40^ or between participants with low versus high illusion rates. These studies have yielded heterogeneous findings. Notably, they have not specifically addressed the VAN but followed a predominantly exploratory approach. A positive deflection peaked at 120 ms over occipital sites, was localized to extrastriate visual cortex, and covaried across participants with susceptibility to the sound-induced flash illusion, whereas on a trial-by-trial basis, illusion perception was associated with enhanced negative deflections peaking at 110 ms (auditory cortex) and 130 ms (superior temporal gyrus^40^). In a follow-up study, a fronto-central negativity at 130–160 ms distinguished illusion from no-illusion trials and was localized to the superior temporal gyrus; the earlier 120 ms occipito-temporal positivity was replicated as a cross-modal interaction effect^41^. A later negativity peaking at 270 ms with a centroparietal maximum appeared in cross-modal interaction difference waves for both illusory and veridical second flash conditions^40^. Additionally, an event-related difference at 265–280 ms, source-localized to the cingulate cortex, differentiated illusion from no-illusion trials^42^. In a TIFI paradigm, an occipital difference between 140 and 185 ms distinguished illusion from no-illusion trials^35^, indicating VAN-like spatial and temporal properties.

Beyond evoked potentials, oscillatory dynamics, particularly in the alpha band, are central to shaping perceptual outcomes, modulating excitability and perceptual thresholds^42‚48^. Alpha activity has also been linked to illusory perception. In auditory paradigms like speech-in-noise tasks, illusion trials are associated with reduced alpha power over temporal regions, reflecting heightened cortical excitability^49^. However, this is not consistent across paradigms: Faramarzi et al.^32^ found no pre-stimulus alpha differences between illusory and non-illusory auditory trials. In SIFI and TIFI paradigms, alpha-band dynamics show consistent correlations with illusion susceptibility. Lower individual alpha frequency^50,51^, reduced pre-stimulus alpha power^52^, and specific alpha phase alignments^35^ have each been associated with a higher likelihood of illusion perception (for review see ^53^).

To assess subjective illusion perception, it is essential to provide participants with a means of reporting their conscious experience. The Perceptual Awareness Scale (PAS) offers a sensitive and theoretically grounded approach for capturing gradations in illusion perception. It distinguishes between varying levels of conscious access and has been shown to align well with both behavioral and electrophysiological measures^54–57^. Notably, there is an ongoing debate as to whether consciousness unfolds gradually^54,56–61^, or appears in an all-or-none manner^62,63^. This ongoing controversy underscores the relevance of nuanced tools like PAS for mapping the structure of conscious experience.

The current study employed an adapted SIFI paradigm to reliably elicit illusory percepts in approximately 50% of trials. Leveraging a large sample size and fine-grained subjective reports, this study aims to determine whether VAN-like negativities can also be observed for consciously perceived illusory stimuli, i.e., in the absence of physical visual input, and whether these correlate with graded levels or dichotomous states of reported consciousness. In addition, the study tests whether pre-stimulus alpha-band activity, specifically its power, predicts the likelihood of illusion perception.

## Materials and methods

### Participants

Sixty right-handed volunteers between 18 and 30 years old (female = 49; male = 10; divers = 1) were recruited at the University of Muenster to participate in the EEG experiment. An a-priori sample size calculation was on one hand not feasible due to expected inter-individual variability in illusion susceptibility and on the other hand incompatible with the planned cluster-based permutation (CBP^64^) analysis. Anticipating relatively small effect sizes and accounting for an estimated dropout rate of 10% and expected data loss (e.g., due to artifact rejection and behavioral exclusion), we selected the largest practicable sample size available, resulting in the above mentioned recruited sample of *N* = 60. All participants had normal or corrected-to-normal vision and no history of psychiatric or neurological diseases. Participants provided written informed consent and received monetary compensation of 12 euros per hour. One participant was excluded because their behavioral data indicated that they were unable to distinguish between one or two flashes presented without auditory stimuli (*d’* = 0.35). Furthermore, 12 subjects were excluded because they did not provide enough trials to obtain reliable ERPs (see section EEG data analysis). This resulted in the final number of *N* = 47 participants (female = 38; male = 8; divers = 1: *M* = 23.13 years, *SD* = 2.39 years) who were included in the main statistical analysis. The study was approved by and conducted following the guidelines of the ethics committee of the local medical association (Ärztekammer Westfalen-Lippe; 2019-049-f-S).

### Apparatus

The experiment was run with Matlab (R2021a, Mathworks Inc., Natick, MA; http://www.mathworks.com) and the Psychophysics toolbox^65,66^. Stimuli were displayed on an LCD monitor (Iiyama G-Master GB2488HSU; 1920 × 1080 pixels, 60 Hz) on a black background (0.35 cd/m^2^) in a dimly lit room. Participants were seated at a viewing distance of ≈ 60 cm in front of the computer. A chin rest was used to prevent head movements during the experiment. Auditory stimuli were presented via two loudspeakers, positioned to the left and right of the monitor.

### Stimuli and presentation

**Figure 1:**
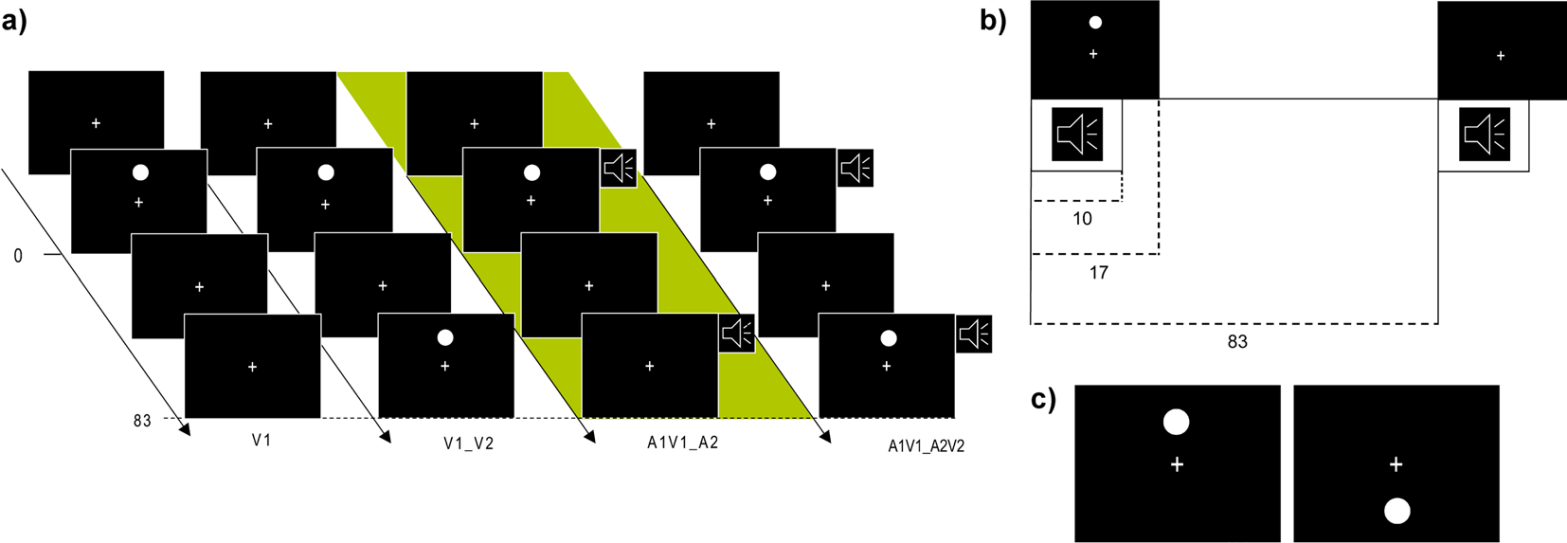
Stimulus Configuration and Experimental Design **(a)** Trial sequence and stimulus parameters. Four randomly presented combinations of auditory (A) and visual (V) stimuli, 1 = fist, 2 = second stimulus. **(b)** Stimulus onset asynchrony (SOA) and temporal configuration. The speaker icon indicates a 10 ms auditory stimulus; the white disk´s duration was 17 ms. Both sensory stimuli had the same onset. The SOA between first and second stimulus presentation is 83 ms. **(c)** Overview of the experimental design. The spatial configuration of the visual stimulus is presented. A white disk(s) were positioned either above (upper visual field, UVF) or below (lower visual filed LVF) the fixation cross. Figure 1a-c is not to scale.

Our EEG study was based on the SIFI paradigm to induce an illusion and then analyze its neural correlates. Therefore, we used two types of stimuli: Auditory (A) stimuli consisted of 1000 Hz sinusoidal beeps, lasting 10 ms, at 80 dB SPL (measured at mid chinrest position, horizontally centered and approximately at mid-head height). Simultaneously, visual (V) stimuli were presented as white flashes, appearing as discs with a 1° visual diameter and a luminance of 330 cd/m². Each flash was presented for 17 ms (Figure 1b). As peripheral presentation of visual stimuli increases the likelihood of successfully eliciting illusions compared to centrally presented triggers^33,37^, the discs were positioned either in the lower visual field (LVF) or in the upper visual field (UVF; Figure 1c) in a random order, at an eccentricity of 6° from the central fixation.

## Experimental design and statistical analysis

### Procedure

Participants were instructed beforehand, emphasizing that their responses should reflect their personal, initial visual impressions and spontaneous decisions, rather than deliberating on what might be considered “right” or “wrong.” The main experiment lasted approximately 1 h and consisted of 960 trials in total, divided into 12 blocks, each containing 80 trials. Every block was separated by breaks to relax the eyes and to get informed about how many trials they had already completed out of the total number. By pressing a key, they were able to proceed with the experiment on their own terms. Four different stimulus combinations were randomly presented (Figure 1a) each occurring 240 times (120 UVF, 120 LVF). The combinations included unimodal visual stimuli appearing singly (V1) or in pairs (V1V2) and bimodal combinations: A1V1_A2V2 and A1V1_A2 (= illusion condition, see definition-below). The stimulus onset asynchrony (SOA) was set at 83 ms. Each trial started with a fixation cross (randomized duration between 1 and 1.5 s), followed by one of the above-described stimulus combinations. Participants were asked to watch the cross permanently. After each trial, the subjects were prompted to report the visibility of the second flash with a 600 ms delay after the stimulus offset (in order to minimize motor-related activity during the analysis of ERPs).

For the reports, we used the perceptual awareness scale to measure the subjective perception as sensitively as possible^67,68^. In pilot experiments, we asked three observers to verbalize their percept on each trial. This procedure led to an adapted 4-leveled scale, closely resembling the scale labels^67,68^. The participants were instructed to use the PAS by pressing the keys 1 to 4 on a standard keyboard with the following adapted labels: 1 = “*no experience of a second flash*”, 2 = “*vague experience of a second flash*”, 3 = “*almost clear experience of a second flash*”, 4 = “*clear experience of a second flash*”. Prior to the main experiment, 32-trial practice sessions were conducted to familiarize participants with the possible perceptual outcomes and the corresponding PAS levels.

### Behavioral data analysis

To obtain a measure of the individual ability to discriminate the presentation of one and two flashes (i.e. conditions V1 and V1V2), we dichotomized the PAS responses, relabeling PAS = 1 as “no (second flash)” and PAS > 1 as “yes (second flash)”. Based on the relative proportions of hits (p(“yes” | V1V2)) and correct rejections, (p(“no” | V1)), we calculated the signal detection theory-based index d’^69,70^. To quantify the effect of auditory stimulation on the frequency of illusory perception of a second flash, we calculated the difference between the relative frequencies p(“yes” | V1) and p(“yes” | A1V1_A2).

### EEG recording and preprocessing

Electrophysiological data were collected using a 64-channel BioSemi active electrode system (BioSemi B.V., Amsterdam, Netherlands). Electrodes were positioned according to the extended international 10–20 system as implemented in the BioSemi 64-channel cap. The BioSemi EEG system replaces traditional ground and reference electrodes with a CMS/DRL feedback loop, using two additional electrodes. Vertical eye movements (VEOG) were recorded with electrodes placed above and below the left eye, while horizontal eye movements (HEOG) were captured with electrodes at the outer canthi of both eyes. Electrical potentials were recorded, maintaining electrode offsets below 20 µV.

EEG data preprocessing was performed using Brain Electrical Source Analysis (BESA) Research 6.0 (BESA GmbH, Gräfeling, Germany). Offline data were re-referenced to an average reference and filtered with a 0.01 Hz high-pass zero-phase filter (12 dB/oct) and a 40 Hz low-pass zero-phase filter (24 dB/oct). A 50 Hz notch filter was applied to remove line noise. Channels with extreme noise were identified by eye inspection, i.e., deflections that appeared to be uncorrelated with neighboring channels and whose amplitudes were roughly at least three times higher than the remaining channels. Those channels were subsequently interpolated (number of interpolated channels ≤ 13; *M* = 4.74, *SD* = 5.66). Eye movements were corrected with BESA’s automatic eye-artifact correction method^71^. Additional artifacts were removed using BESA’s semi-automatic, principal component analysis (PCA) based artifact topography algorithm, with manual selection of artifacts. Trials exhibiting muscle artifacts, electrode jumps, or amplitudes exceeding a 100 µV threshold were rejected following visual inspection.

The main analysis focused on the comparison of trials with and without illusions (henceforth called ‘seen’ and ‘unseen’, referring to the second flash) in the critical condition, i.e., A1V1_A2. The proportion of illusion trials can vary across subjects^51,72,73^. Thus, in our experiment some subjects may provide unreliable ERPs for either the seen or unseen condition if they are based on a low number of trials. While including only subjects with a high number of trials optimizes the reliability of individual ERPs, it reduces the overall sample size and, thus, the reliability of the grand average ERP. To optimize this trade-off, we chose a data-driven, hypothesis-independent approach and systematically evaluated the signal-to-noise ratio (SNR) of the critical condition, i.e., averaged across seen and unseen trials, for a range of minimum trial numbers (between 10 and 100 in steps of 5). To be included in the final sample, the number of seen and the number of unseen trials in the critical condition had to match or exceed this number. For each minimum trial count, we calculated the root mean square (RMS) of the averaged EEG signal. SNR was derived as the ratio of RMS signal during the critical time window (0–300 ms) to the baseline period (−200-0 ms). The highest SNR was observed at 30 trials, leading to the exclusion of the above-mentioned 12 participants. The average number of analyzed trials in the final sample in the critical conditions was mean_unseen_ = 120.96 (*SD* = 46.53) and mean_seen_ = 106.89 (*SD* = 45.51).

Illusory percepts were also expected to occur without auditory stimulation, i.e., in the V1 condition. These illusions were expected to be less frequent, as the participants’ expectation of a second flash was not influenced by the auditory stimulus but by the perceived probability of the appearance of a second flash. We also note that a proportion of these reports could arise from accidental button presses or lapses rather than genuine illusory percepts. To validate possible VAN effects observed in the critical condition, we performed a comparison for seen vs. unseen second flash also in the V1 condition. We applied the same SNR-optimization approach also for this comparison, which led to the exclusion of 26 participants. The average number of trials analyzed in the final for the control condition was mean_unseen_ = 148.09 (SD = 41.34) and mean_seen_ = 80.12 (SD = 38.21).

### EEG data analysis

Intervals and electrodes of interest were defined based on previous studies to optimize the detection of electrodes maximally responsive to the VAN. We considered all posterior and occipital electrodes, as supported by prior research (e.g.^7,8,74,75^), which indicates that these regions show heightened negativity linked to early conscious visual processing. Prior studies ^3,5‚76‚77^ suggest that the typical VAN time window falls somewhere between 100 and 300 ms. In our study, we focused on VAN effects following the second (illusory) flash, occurring 80 ms after the first flash onset. Consequently, we expected the VAN response to appear around 180–380 ms post the initial flash onset in the critical condition (A1V1_A2). Within the defined interval and electrodes of interest, we employed a CBP^64^. CBP tests were performed by computing a paired-sample t-test at each channel and sample of interest. The cluster mass was calculated by summing all t-values with alpha < 0.05 (one-sided negative) neighboring in time and space (the minimum number of channels to form a cluster was 2). The number of permutations was *N* = 5000 and the significance threshold for testing the null hypothesis was alpha < 0.05. The interval of interest extended from 180 to 380 ms and the channels of interest included P1, P3, P5, P7, P9, PO7, PO3, O1, Iz, Oz, POz, Pz, P2, P4, P6, P8, P10, PO8, PO4, and O2. Secondary analyses and visualization of ERPs were conducted using electrodes identified as significant, i.e., all channels with at least one significant sample. Additionally, we explored whether the VAN followed a gradual or dichotomous pattern across the four levels of the PAS scale, using the cluster average, i.e., the mean value of the significant sensors and samples described by the cluster as a measure of the VAN amplitude. We then compared Linear Mixed-Effects Models (LME) with single-trial VAN amplitudes as the dependent variable (DV). All analyses were conducted in R, utilizing the lme4 package^78^ for model building and the readr package for data handling and preprocessing. Only trials from the critical experimental condition were retained for analysis. The DV was centered and scaled to facilitate the interpretation of model parameters. To evaluate gradual effects, perceptual awareness was decomposed into orthogonal polynomial contrasts, capturing linear, quadratic, and cubic trends^57^. A baseline model was constructed with only a random intercept for participants to account for individual differences in VAN amplitude. Subsequent models incorporated predictors reflecting either gradual changes in perceptual awareness or dichotomous perceptual outcomes. To test for a dichotomous pattern of awareness, a fixed effects model was specified using a categorical predictor that indexed trials as either illusion or no-illusion, despite identical physical stimulation. This model included a fixed effect for the dichotomous predictor and a random intercept for participant ID. Fixed effects models were selected because models with random slopes failed to converge. Likelihood ratio tests were used to compare model fits, allowing us to assess whether variability in VAN amplitude was better accounted for by gradual or dichotomous patterns of perceptual awareness.

### Spectral analysis

Power spectra were computed using a Fast Fourier Transform of single-trial raw data in the 500 ms pre-stimulus time window. Power was tested for differences between illusion and no-illusion trials in the alpha frequency range (8 to 12 Hz), averaged across three channels, which showed the strongest overall alpha power (PO3, POz, PO4).

## Results

### Behavioral data

#### Distribution of awareness ratings

**Figure 2:**
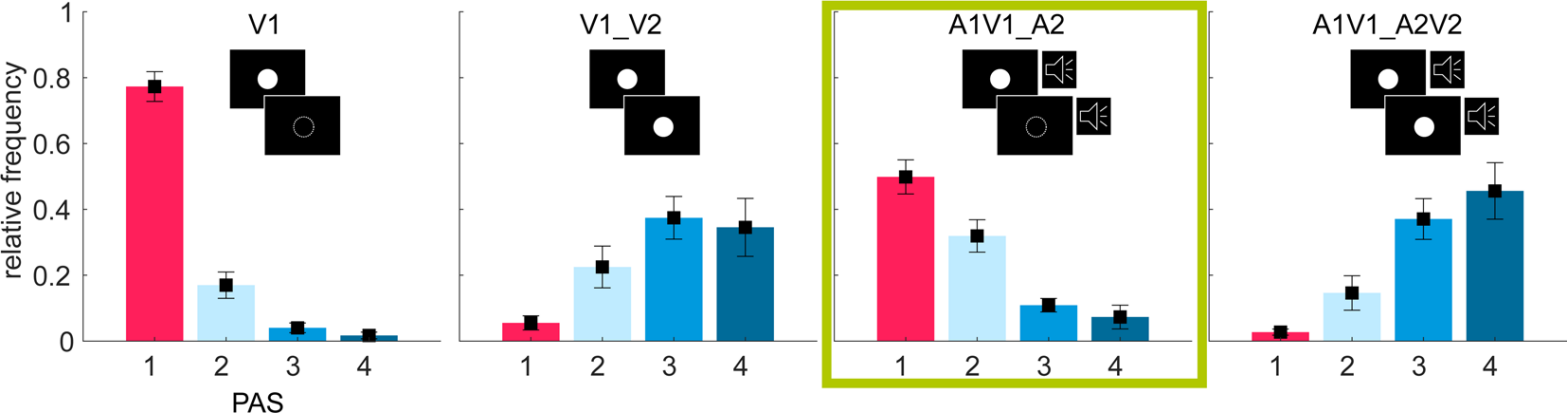
PAS Ratings and response probabilities for all conditions. Higher PAS ratings indicate clearer perception of the second stimulus. V = Visual stimulus; A = Auditory stimulus; 1 = first stimulus; 2 = second stimulus; PAS = Perceptual Awareness Scale (1 = no perception of the second stimulus, 2 = vague perception, 3 = almost clear perception, 4 = clear perception).

PAS Ratings for all experimental conditions are shown in Figure 2. In 20.98% (*SD* = 0.154) of trials in the V1 condition, participants reported perceiving a second flash (PAS > 1) despite its physical absence, which allowed us to use this condition as a control comparison. A one-way repeated-measures ANOVA revealed a significant main effect of condition on the relative frequency of PAS1 responses, F(3, 184) = 315.059, *p* <.001, η² = 0.855, indicating that the frequency of perceiving only one flash varied substantially across conditions. As shown in Table 1, PAS1 responses (i.e., *“no second flash perceived”*) were most frequent in the unimodal visual condition (V1) and least frequent in the multisensory congruent condition (A1V1_A2V2).

**Table 1:** Relative frequency of PAS1 responses per condition.

Condition	Mean (M)	SD
V1	0.7902	0.1539
V1_V2	0.0571	0.0711
A1V1_A2	0.0274	0.0306
A1V1_A2V2	0.4733	0.1979

Pairwise comparisons (Table 2) confirmed statistically significant differences between all conditions (*p* <.001). Notably, the comparison between the two bimodal conditions (illusion vs. veridical: A1V1_V2 vs. A1V1_A2V2) revealed a significant difference, t(46) = 4.58, *p* <.001, d = 0.59.

**Table 2:** Pairwise comparisons of PAS1 frequencies between conditions.

Comparison	t(59)	*p*-value	Mean Difference	Cohen’s d
V1 vs. V1_V2	34.9619	< 0.001	0.7331	5.0997
V1 vs. A1V1_A2V2	34.6793	< 0.001	0.7628	5.0585
V1 vs. A1V1_A2	7.1170	< 0.001	0.3168	1.0381
V1_V2 vs. A1V1_A2V2	3.6954	< 0.001	0.0297	0.5390
V1_V2 vs. A1V1_A2	−12.3706	< 0.001	−0.4162	−1.8044
A1V1_A2V2 vs. A1V1_A2	−14.4465	< 0.001	−0.4459	−2.1072

### Electroencephalography data

**Figure 3:**
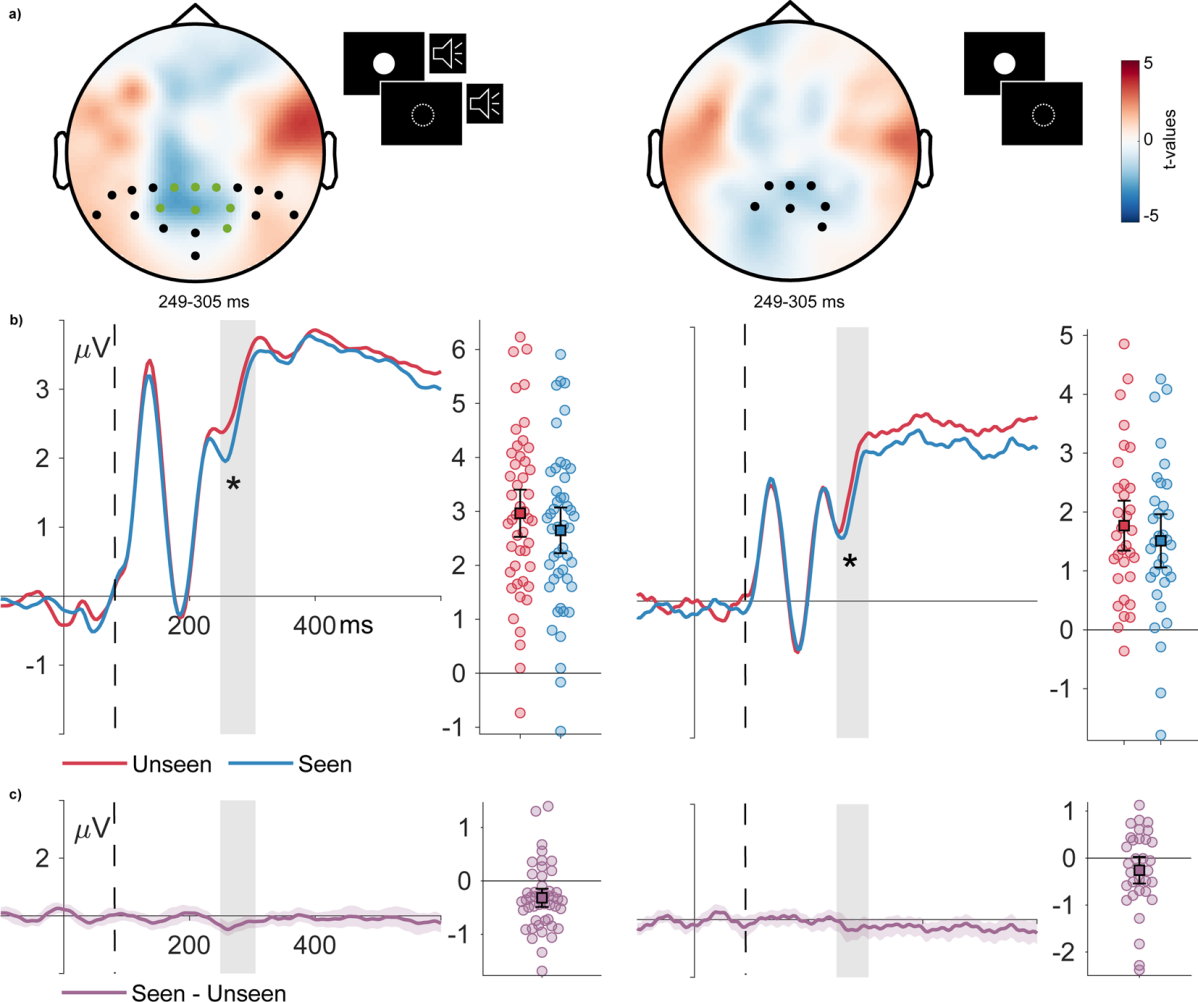
Topography and ERPs for the seen vs. unseen difference in the critical and control condition **(a)** Topographic maps for the critical condition (A1V2, left) and control condition (V1, right) illustrate the spatiotemporal expression of VAN between 250 to 305 ms for seen versus unseen stimuli over bilateral occipito-temporal sites, with significant clusters (in green) indicating greater negativity for seen stimuli. **(b)** Electrophysiological results in the critical condition A1V1_A2 and control condition V1: ERPs for seen (PAS > 1, blue) and unseen (PAS = 1, red) trials. **(c)** Difference waves visualize the difference between seen - unseen trials. The shaded area around these difference ERPs indicates the 95% bootstrap confidence interval. Error bar plots present the average amplitude at selected sensors and intervals of interest, with a 95% confidence interval. Dashed line marks the second stimulus´ onset.

As noted above, a sample of *n =* 33 remained after applying the criterion of a minimal number of 30 seen and 30 unseen trials for this control comparison. The main analysis is based on comparisons between seen and unseen trials in the critical condition (A1V1_A2). Figure 3 (left) shows the corresponding ERPs and the differential topography. A control analysis is presented for the comparisons between seen and unseen trials in the V1 condition. Corresponding ERPs and topographies are depicted in Figure 3 (right). ERPs to the four experimental conditions without post-hoc sorting into seen and unseen trials are presented in the Supplementary Material.

### Visual awareness negativity - seen vs. unseen comparison

For the critical condition (A1V1_A2), we observed a significant cluster in the interval and channels of interest (cluster mass = −167.765, *p* =.016). Descriptively, the cluster extended from approximately 250 to 305 ms, as shown in Figure 3, which depicts the ERPs averaged across all significant electrodes (P1, Pz, P2, PO3, POz, PO4, and O2). Since CBP tests do not precisely establish the latency and location of effects^79^, the spatiotemporal extents of clusters are provided only for descriptive purposes. On the right side in Figure 3, we also provide results for the control condition (V1) for the comparison of trials with and without the illusory perception of a second flash. In both conditions, the VAN can be shown in the expected time window.

The topographies in Figure 3a visualize the spatiotemporal dynamics of the VAN within the significant time window of 250–305 ms where seen stimuli exhibit greater negativity compared to unseen stimuli. The significant cluster of electrodes is marked in green for the critical condition. The topographies for both conditions show a negativity predominantly localized over posterior-central sites. We tested whether VAN in the critical condition was affected by upper or lower presentation using a Bayesian paired t-test, to determine whether presentation location influenced VAN amplitudes. The results confirmed that there was moderate evidence for the null hypothesis (BF01 = 4.9453) i.e., for no VAN differences between the upper and lower hemifield.

### Gradual vs. dichotomous correlates

**Figure 4:**
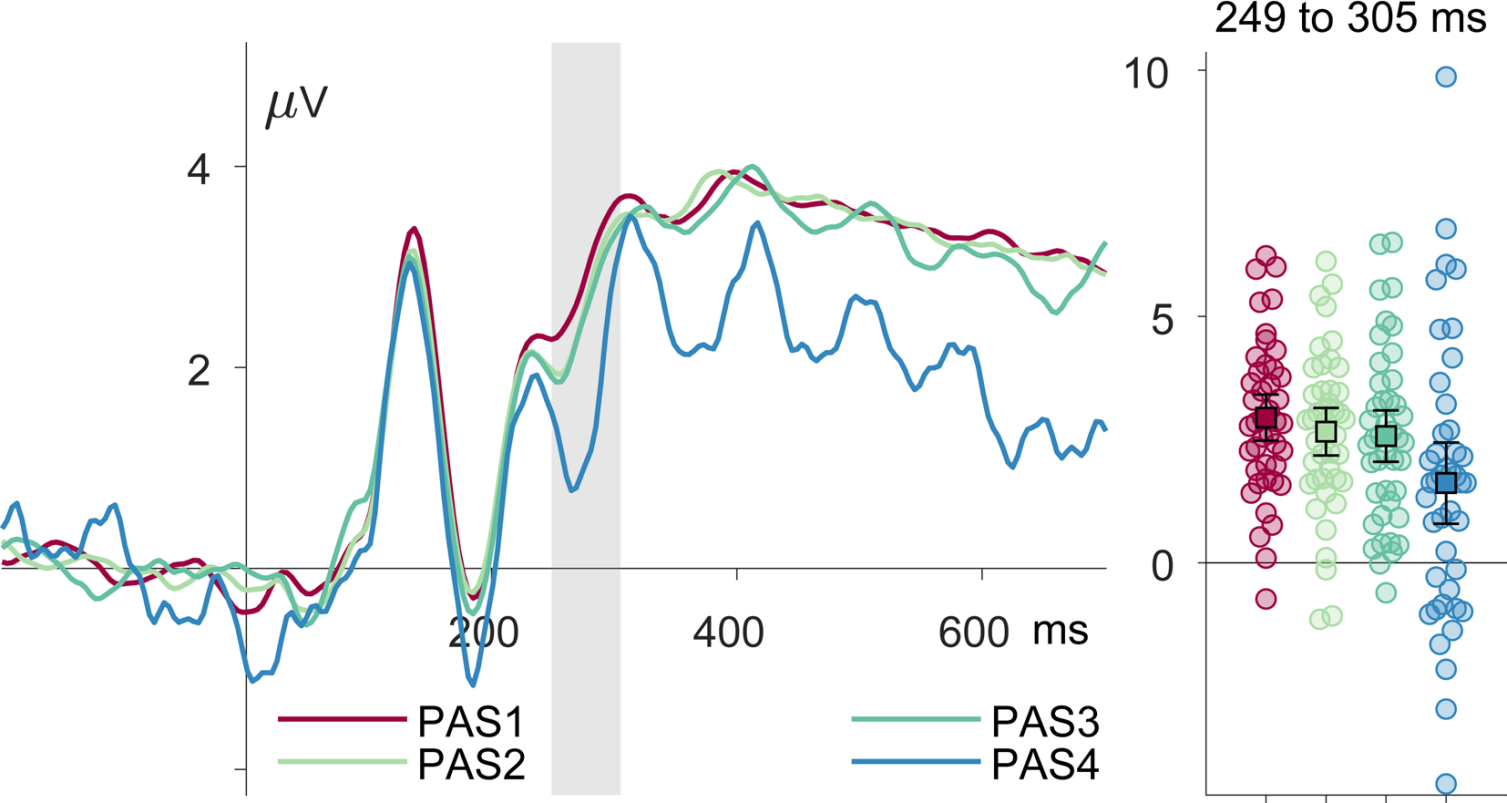
PAS Levels in the Critical Condition (*N* = 38). ERPs for four PAS categories from PAS1 to PAS 4 (1 = no perception of the second stimulus, 2 = vague perception, 3 = almost clear perception, 4 = clear perception) in the critical condition A1V1_A2. The left panel depicts the ERP with color-coded PAS levels red: PAS 1, light green: PAS 2, dark green: PAS 3, blue: PAS 4, while the right panel shows mean amplitudes with error bars for each PAS level over the 250–305 ms time window.

Both linear mixed-effects model treating PAS as a gradual or a dichotomous (see vs. unseen) predictor of VAN amplitude significantly outperformed the null model, (χ²(1) = 6.94, *p* =.008 for the graded and χ²(1) = 5.52, *p* =.019, for the binary model. However, PAS showed only a marginally better fit than detection (AIC: − 17356 vs. − 17355; BIC: − 17327 vs. − 17326), which does not indicate a meaningful statistical difference in overall model fit. Thus, while both Figure 4 and statistical results suggest at least descriptively a gradual component in the VAN response, our data allow no firm conclusions.

### Exploratory analysis of pre-stimulus alpha power

**Figure 5:**
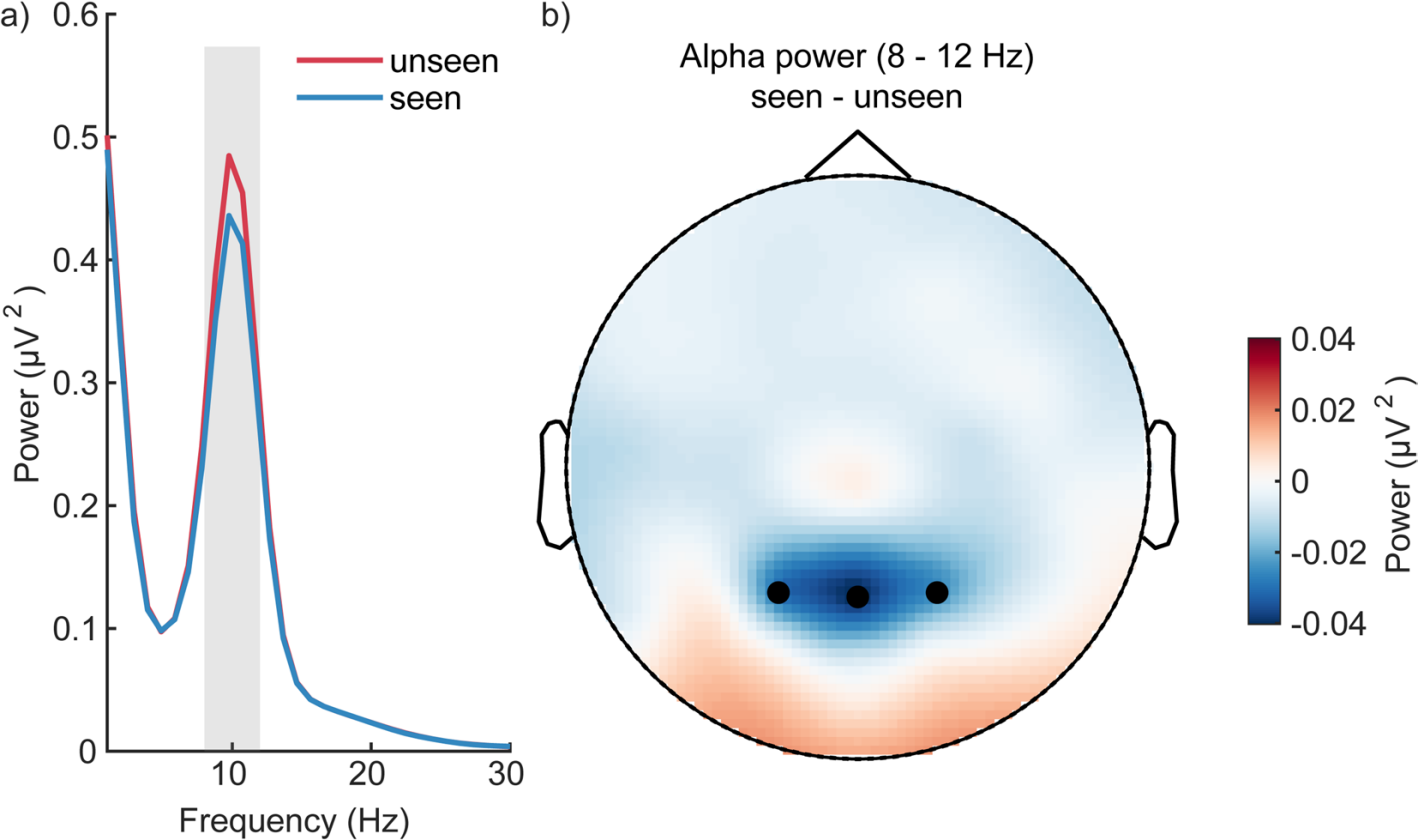
Spectral analysis of pre-stimulus alpha power. (**a**) Mean alpha-band power is plotted separately for illusion (seen) and no-illusion (unseen) trials. (**b**) Topography of the difference (seen – unseen) in alpha power (8–12 Hz).

Alpha-band power in the pre-stimulus interval was stronger preceding trials without illusions compared to trials with illusion (t(46) = 2.32; *p* =.025; two tailed; Figure 5).

## Discussion

We analyzed whether the VAN could serve as an NCC even in the absence of direct sensory stimulation. We used the SIFI paradigm to examine whether the VAN would be elicited when participants perceived an illusory second flash compared to non-illusory conditions and found a negativity between 250 and 300 ms, aligning with established VAN characteristics(e.g^7,26,74‚80^). In our study design, illusion can be interpreted within a predictive coding framework, which suggests that strong perceptual priors (shaped by repeated exposure and simultaneous auditory stimuli) can give rise to illusory percepts in the absence of external input^28,81^. Our findings foster the robustness of the VAN as a correlate of subjective visual experience even in illusory perception contexts, supporting the notion that the VAN may reflect conscious perception itself, even when no physical visual input is present.

Our results contrast with accounts that question the VAN’s role in conscious perception and instead link awareness more consistently to later ERP components^20–24^ and with views that interpret the VAN as reflecting early, pre-conscious processing^25,26^. Conversely, other work associates conscious access with both the VAN and a subsequent late positivity^19^. Our findings provide a counterpoint to a purely pre-conscious interpretation: because a VAN-like negative deflection is present during the conscious perception of physically absent stimuli, its generation cannot be reduced to stimulus-driven input alone and may index processes tied to conscious awareness. This interpretation is in accordance with the Recurrent Processing Theory^82,83^, which posits that unconscious feature extraction is mediated by a fast feedforward sweep, whereas conscious percepts arise from recurrent cortico-cortical feedback. Activations in the VAN time window might therefore be associated with constructing a coherent percept. For example, beyond processing simple physical features, the VAN may reflect automatic binding of stimulus representations to the self; early ERPs around 250 ms become larger when stimuli are self-referenced^84^; indicating integration of both egocentric and allocentric representations. Thus, VAN activity could arise from the combined influence of feedback processing and self-related integration, rather than solely from feedforward sensory activation.

Our results are consistent with EEG and MEG studies indicating that illusory and veridical perception can share key neural signatures—especially in oscillatory dynamics and ERP profiles^27,31,32,35,40–42^. In line with previous findings^12^ we assume that the VAN reflects a sensory-level signal within the broader family of Perceptual Awareness Negativities, which have been observed across sensory modalities (AAN^15–18^, VAN^5–9^, SAN^13,14^) and typically emerge over the corresponding modality-specific cortical areas. This interpretation is supported by the posterior distribution and early latency of the VAN, suggesting that it originates from neural activity within visual sensory regions rather than from later supra-modal stages. Most EEG studies on the SIFI address multisensory integration rather than the neural determinants of perceptual awareness. However, a few studies have directly contrasted illusion and no-illusion perception. A fronto-central negativity around 130–160 ms has been shown to distinguish illusion from no-illusion trials, although without the posterior distribution typical of the VAN^41^. An event-related difference at 265–280 ms, source-localized to cingulate cortex, has also been linked to illusion perception, but this effect differs from ours in both timing and scalp topography^42^. In a touch-induced illusion, an occipital difference 140–185 ms distinguished illusion from no-illusion trials^35^ and was thus spatially and temporally similar to our effect. Two previous studies, which induced auditory illusions, also reported negativities as correlates of illusory perception. An ERP interpretable as the auditory-awareness negativity was identified in a study which relied on spontaneous auditory illusions occurring in noise and contrasted trials with and without reported perception, allowing for a direct comparison of perceived versus unperceived auditory events^32^. Furthermore, in a study which used a design with associative learning between cues and auditory stimuli, an AAN was observed over temporal and parietal electrodes between 200 and 300 ms, closely paralleling the visual VAN in timing and distribution^31^. We focused our ERP analysis on posterior scalp sites, encompassing occipital, parieto-occipital, and lateral temporal electrodes (see^3,7^). Within this a-priori space, we detected a robust negative deflection that temporally matches the VAN but peaks over mid-parietal electrodes (P1, Pz, P2, PO3, POz, PO4, O2). Studies show that the VAN amplitude and scalp distribution can shift with stimulus complexity^55,74,85^, perceptual visibility (for review see^86^task relevance (e.g.^87^) and experimental context^7,57,87,88^. Hence, our findings contribute to the growing evidence that VAN (topography) does not seem to be a fixed phenotype but can be modulated by experimental context. Given its timing and predominantly posterior distribution, one plausible interpretation is that the VAN arises at the sensory processing stage. Interestingly, other ERPs (e.g., mismatch negativity^89^ and N400^90^) are consistently linked to additional processing demands when stimulus features violate expectations (physical or semantic), aligning with prediction-error accounts rather than with conscious experience per se^91,92^. As noted in the introduction, an increased VAN for trials with illusions compared to trials without suggests that it does not reflect expectation violations, as these would be larger in trials with the veridically perceived absence of the second flash. However, we suggest that negative-going ERPs in similar early time windows show increased processing of sensory input and can be at least in part an NCC, if the suited contrasts are used.

In addition to ERPs, we examined oscillatory dynamics, particularly alpha-band activity. Other than in an auditory illusion study, which found no pre-stimulus alpha differences between illusion and no-illusion trials^32^, our results are in line with studies showing that lower individual alpha frequency^50,51^, reduced pre-stimulus alpha power^52^, and specific pre-stimulus alpha phase alignments^35^ increase susceptibility to illusion perception in the SIFI and TIFI paradigms (for review see^53^). While these studies interpret al.pha primarily within the framework of temporal precision for multisensory integration, we suggest that alpha frequency may also index the cortical conditions underlying the access of perceptual content to conscious perception, modulating whether internally or externally driven signals gain perceptual access.

For a comprehensive account of the neural marker, the correspondence with participants’ subjective reports is critical. The behavioral data from the V1, V1_V2 and A1V1_A2V2 conditions provide benchmarks, validating participants’ reports across varying sensory contexts. There is reason to consider the PAS ratings in our study as a meaningful reflection of subjective experience, although we cannot rule out that a small proportion of responses were the result of lapses or confused keys. The V1_V2 and A1V1_A2V2 conditions exhibit progressively higher PAS responses where additional sensory information enhances perceptual clarity. In the critical condition, illusions were observed on 50% of trials, congruent with the literature (for review see ^37^). Overall, the resulting distribution of ratings confirms our previous expectations, and in addition to the consistent pattern across conditions, this supports the reliability of the behavioral data in our study. However, we also found that in 24% of V1 trials (where only one flash was presented without any auditory input), participants reported perceiving a second flash, despite the absence of a corresponding stimulus. These illusions are assumed to be caused by strong perceptual priors shaped by the repeated exposure to two consecutive flashes.

In a predictive coding framework^29,30^, the illusions observed in our paradigm, both in the V1 and A1V1_A2 condition, can be explained as a consequence of the expectation that the second flash will appear in combination with a prediction error signal that is too small to trigger an update of this expectation. In contrast to hit-vs-miss paradigms, a large prediction error thus leads to the veridical perception of the absence of a second flash, and a small prediction error causes its illusory perception. Consequently, a larger VAN for trials with illusions compared to trials without is not compatible with the idea that the VAN indexes the prediction error, fostering its interpretation as a correlate of consciousness per se. In our paradigm, one- and two-flash trials were deliberately made difficult to distinguish, introducing residual uncertainty into the system. Under such conditions, the brain integrates prior knowledge with incoming sensory data to minimize prediction error, especially in regions lacking strong afferent stimulation. When predictions closely match incoming input, residual errors can become negligible, allowing early cortical activity to resemble that of veridical perception, even when the stimulus is absent^93,94^. The frequent occurrence of two-flash trials likely strengthened participants’ prior expectation that a second flash would follow the first. This learned association may have contributed to the emergence of double-flash percepts, even when only one flash without auditory input was presented. Illusions in the unimodal V1 condition occurred less frequently than in the multisensory SIFI condition, where the auditory beep further reinforced the “two-flash” prior. Yet the convergence of real and illusory perception has its limits. Participants seem to be able to reliably distinguish between true and illusory percepts^95^. Research on mental imagery demonstrates that while vivid mental images share substantial overlap with veridical perception, they can still be differentiated both behaviorally and in neural activation patterns (for review see^96^). Because in our study, participants might have known the second flash could be illusory (i.e., potentially not real), they may have down-weighted its credibility, reducing the binding of the percept to allocentric self-representations. Such context-sensitive belief effects on stimulus–self binding have been reported in recent work^84^, and similar manipulations of perceived reliability (e.g., telling participants they might be under a psychosis-related drug) have been associated with more negative occipito-temporal ERPs in the same time window compared to controls. Beyond belief effects, an additional consideration is how the early visual cortex resolves competition between a putative second flash and the black background. The percept of a second flash may involve not only activation of a white-disc representation but also suppression of the competing representation of the black background. In this context, increased negativity in the N1 time window over occipito-temporal sites could index the sidelining of incongruent background signals. Notably, central N1 components have been linked to early, automatic inhibition of stimulus-evoked representations^91^. In our data, ERPs for PAS4 show at least descriptively more negative occipito-temporal activity (see Figure 4), which would be consistent with such suppression.

A question that remains vacant is whether conscious perception unfolds gradually or appears in an all-or-none manner. When plotting ERPs for the four PAS categories in the critical condition, visual inspection suggested a gradual pattern of consciousness. Yet, the data seem to lack statistical power to support a statistical difference between the gradual and the dichotomous model. Descriptively, the gradual model shows a slightly better fit, which is in line with several studies^54–56–61^ (but see^62,63^) and supports the importance of suited measurement of graded experience^68^.

We acknowledge several limitations. As outlined above, our goal to gain additional information about the graded vs. dichotomous nature of illusory awareness from the four levels of the PAS could not be reached despite a large number of trials and participants. While the PAS model demonstrated a descriptive advantage over the binary detection model in predicting VAN amplitude, the lack of a statistically significant difference prevents any conclusions, and future studies with more power are needed. Furthermore, we used a specific experimental design to induce illusory percepts. It would be helpful to include different designs in future studies in order to provide data on weather results can be generalized across designs.

## Conclusion

Our findings demonstrate that VAN-like negativities can emerge in response to subjectively perceived visual stimuli, even in the absence of external stimulation. This challenges interpretations of the VAN as purely stimulus-driven and strengthens its candidacy as a neural correlate of visual awareness, and illustrates how studies on visual illusions can inform the research on NCC.

## Supplementary Information

Below is the link to the electronic supplementary material.

Supplementary Material 1

## Data Availability

The datasets analysed during the current study are available from the corresponding author on reasonable request.
