# Supplementary material for: Neural correlates of consciousness in the sound-induced flash illusion
Source: Sci Rep. 2025 Nov 28;15:43109. doi: 10.1038/s41598-025-28068-5 (PMC12678526; doi:10.1038/s41598-025-28068-5)
Supplement: Supplementary file 1 — Supplementary Material 1 [file 41598_2025_28068_MOESM1_ESM.pdf]

Supplementary Materials

for

More than meets the eye: neural correlates of consciousness in the sound-induced flash illusion

Theresa Rieger[1,2+,*], Josefine Feuerstein[1+], Niko A. Busch[2,3], Thomas Straube[1,2] & Maximilian Bruchmann[1,2]

[1] Institute of Medical Psychology and Systems Neuroscience, University of Muenster
[2] Otto Creutzfeldt Center for Cognitive and Behavioral Neuroscience, University of Muenster
[3] Institute of Psychology, University of Muenster
[+] these authors contributed equally to this work

* Corresponding author: Theresa Rieger

Correspondence address:

Institute of Medical Psychology and Systems Neuroscience
University of Münster
Von-Esmarch-Str. 52, D-48149 Münster, Germany
e-mail: theresa.rieger@uni-muenster.de

Data exploration for all experimental conditions

To appropriately contextualize the ERPs of a second illusory stimulus, it seems useful to first establish an overview of the general ERPs elicited by a second flash in this paradigm. Topographic time series were plotted for all conditions (Figure 3A).

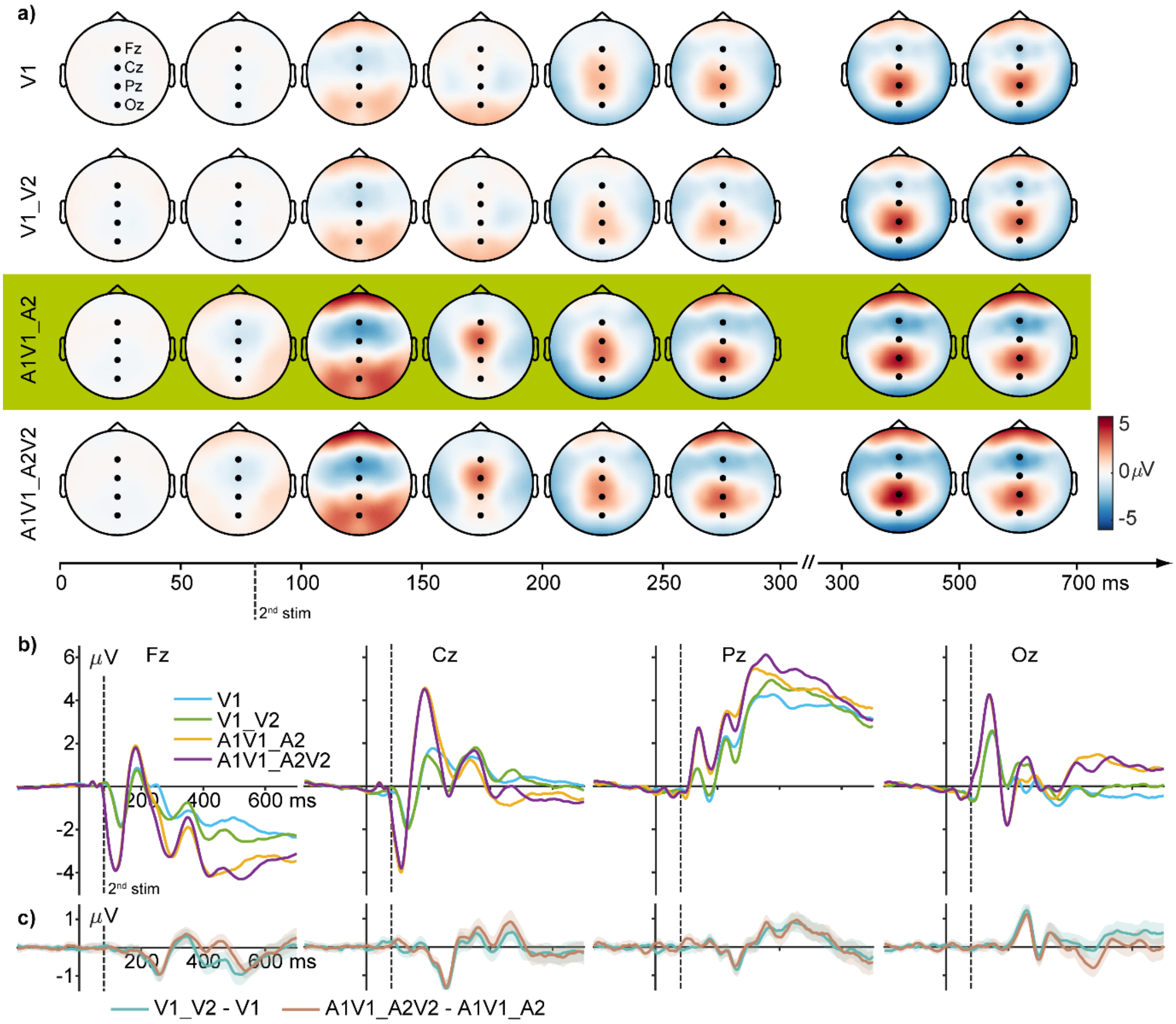

Supplementary Figure S1. Spatiotemporal dynamics and neural signatures across experimental conditions **(a)** Topographic time series for all conditions. Scalp distribution of electrical potential. V = Visual stimulus; A = Auditory stimulus; 1 = first stimulus; 2 = second stimulus. The time series ranges from 0 to 700 ms post-stimulus onset. Time steps are 50 ms from 0-300 ms and then larger (200 ms 300-700 ms). Positive potentials are represented by warmer colors (e.g., red/orange), while negative potentials are represented by cooler colors (e.g., light/dark blue). Exemplary electrodes Fz, Cz, Pz, Oz. **(b)** Related potentials per condition and neural response to a second stimulus. ERPs recorded at four representative sites (Fz, Pz, Cz, and Oz) for different conditions (blue: V1_0, green: V1_V2, yellow: A1V1_A2, and purple: A1V1_A2V2). **(c)** Difference waveforms (right subplots) highlight the contrasts between V1_V2 - V1_0 and A1V1_A2V2 - A1V1_A2 conditions, providing insights into neural responses to the second stimulus (green: visual condition, brown: bimodal condition). Dashed line marks the second stimulus´ onset.

These visualizations provide an overview of the spatiotemporal ERP patterns. For broader insights into the electrophysiological effects of the current paradigm, Supplement Figure S1 illustrates the ERPs from four representative electrodes (Fz, Cz, Pz, Oz) across all conditions V1_0, V1_V2, A1V1_A2, and A1V1_A2V2. The difference waves (see Figure 3C) represent the ERP of the second flash alone, as the effects of the initial visual stimulus have been subtracted (depicted in green for visual conditions and brown for bimodal conditions).
